# Unmasking SMLR1: The hidden player in colorectal cancer’s liver metastasis

**DOI:** 10.47248/chp2401010002

**Published:** 2024-07-18

**Authors:** Xiaozhuo Liu

**Affiliations:** Department of Pharmacology and Therapeutics, Roswell Park Comprehensive Cancer Center, Buffalo, NY 14263, USA

Exploring cancer cell adaptability and the complexity of the tumor microenvironment (TME) is both fascinating and crucial in cancer research. During metastasis, cancer cells must undergo significant changes to adapt and survive in new environment. For colorectal cancer, the liver is a primary site of metastasis. Understanding why certain cancers preferentially spread to specific organs, such as the liver, can provide insights into tumor biology and inform new therapeutic strategies.

In this inaugural issue of *Cancer Heterogeneity and Plasticity*, Wong and colleagues present novel roles of small leucine-rich protein 1 (SMLR1) in colorectal cancer liver metastasis (CRLM). By demonstrating that SMLR1 is upregulated in CRLM and interacts with tumor-associated macrophages (TAMs) via MRC1 (CD206) and SIGLEC1 (CD169), this research unveils a novel mechanism of immune evasion and tumor progression. Notably, SMLR1 upregulation was observed specifically in the CRC liver metastases but not in CRC metastases in other tissues such as the peritoneum (see Figure 1), suggesting a context-dependent interaction between cancer cells and the liver microenvironment that facilitates metastasis.

This study aligns well with the mission of *Cancer Heterogeneity and Plasticity* by exploring the dynamic realm of cancer cell heterogeneity and the complexity and cellular cross-talks in the TME. The elucidation of SMLR1’s role in immune evasion and metastasis contributes valuable insights into cancer cell plasticity and reprogramming. The focus on specific molecular interactions in the liver microenvironment exemplifies the type of research that can drive forward our understanding of cancer adaptability.

While the study provides significant insights, there are areas that require further exploration. The mechanisms underlying SMLR1 upregulation in CRLM remain unclear and warrant additional investigation. Moreover, the therapeutic potential of targeting SMLR1 should be explored *in vivo* to assess efficacy and safety. It would also be interesting to investigate whether lung metastasis exhibits a similar pattern of SMLR1 upregulation and interaction with the microenvironment as seen in liver metastasis or if it resembles peritoneum metastasis. Future studies could broaden the scope of clinical relevance by examining SMLR1 upregulation in other metastatic sites and cancer types.

## Figures and Tables

**Figure 1.**
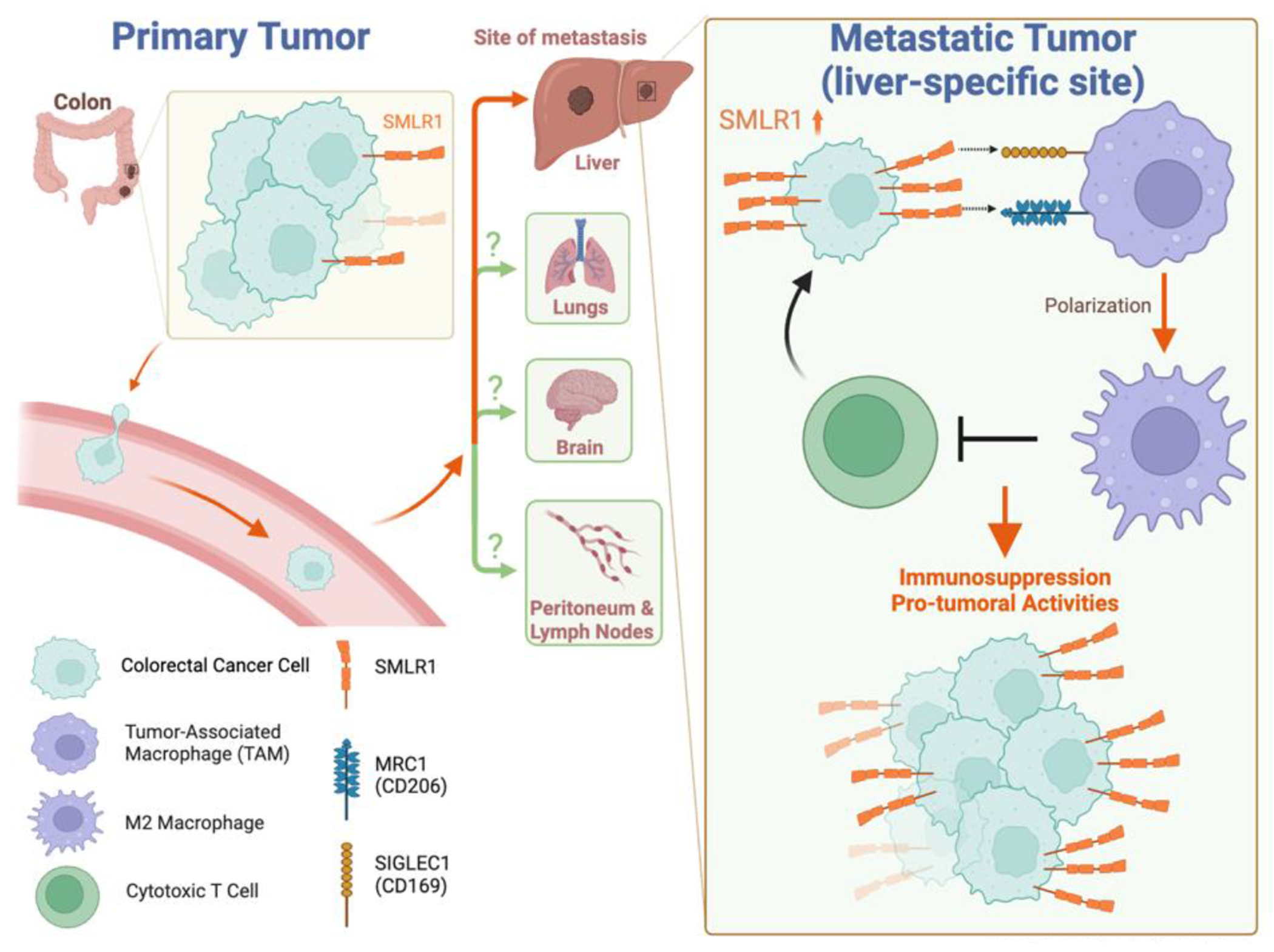
The role of SMLR1 in modulating immune suppression in colorectal cancer liver metastasis. This diagram depicts the elevated expression of SMLR1 in colorectal cancer liver metastases and its strategic interactions with tumor-associated macrophages (TAMs). SMLR1 enhances immune evasion by engaging MRC1 (CD206) and SIGLEC1 (CD169) on TAMs, leading to their polarization toward an M2-like, pro-tumoral phenotype, which contributes to a suppressive tumor microenvironment in the liver. These interactions underline a novel pathway for potential therapeutic targeting to mitigate metastatic progression in colorectal cancer.

## Data Availability

Not applicable.
